# Complete Genome Sequence of *Mycoplasma yeatsii* Strain GM274B (ATCC 43094)

**DOI:** 10.1128/genomeA.00328-15

**Published:** 2015-04-23

**Authors:** Michael J. Calcutt, Bethany N. Kent, Mark F. Foecking

**Affiliations:** Department of Veterinary Pathobiology, University of Missouri, Columbia, Missouri, USA

## Abstract

*Mycoplasma yeatsii* is a goat mycoplasma species that, although an obligate parasite, accommodates this lifestyle as an inapparent commensalist. High-frequency transformation has also been reported for this species. The complete 895,051-bp genome sequence of strain GM274B has been determined, enabling an analysis of the features of this potential cloning host.

## GENOME ANNOUNCEMENT

*Mycoplasma yeatsii* together with *Mycoplasma cottewii* and *Mycoplasma putrefaciens* represent a mycoplasmal clade that is closely connected phylogenetically to the *M. mycoides* cluster of ruminant pathogens (1). Unlike the latter group of agriculturally important taxa, *M. yeatsii* and *M. cottewii* are most commonly isolated as commensals from the caprine ear canal (2). *M. yeatsii* is also of interest, as it is one of only a few mycoplasma species for which both plasmids and successful transformation have been reported (3, 4). To delineate the genetic background of this potential cloning host and to gain further insight into the evolution of the extended *M. mycoides* cluster, the complete genome sequence was determined for the plasmid-free strain GM274B. This isolate was recovered from a Toggenburg goat in California (5).

Initially, genomic DNA was prepared from strain GM274B (obtained as ATCC 43094), and sequenced by 454 chemistry at the Genome Institute, Washington University (St. Louis, MO, USA). Upon assembly (Newbler), this data set yielded 40 contigs (120-fold read depth), largely due to the presence of repeated sequences such as insertion sequences (ISs). Subsequently, an aliquot of the same DNA preparation was sequenced at the National Center for Genomics Research (Santa Fe, NM, USA), using the Pacific Biosciences platform. Reads from a single SMRT cell were assembled by HGAP version 2 (6) into a single contig (273-fold coverage) that was colinear with each of the large contigs generated from the 454 reads. The 895,051-bp genome sequence was automatically annotated using the PGAP pipeline at NCBI, following which the resulting open reading frames (ORFs) were manually curated. The sequences of 25 truncated, disrupted, or point mutation–containing pseudogenes were independently verified.

The resulting annotation represents a genome comprising 792 genes (728 ORFs and 25 pseudogenes), including those for 30 tRNAs and six rRNAs (encoded in two 16S-23S-5S rRNA operons). The G+C content is 25.74%.

While this work was in progress, the genome sequence of *M. yeatsii* strain 13926 was determined (7). This 896,612-bp data set comprises 57 contigs that are largely collinear with the GM274B chromosome. Among the regions of difference are strain variable patterns of IS integration and a 28,334-bp integrative conjugative element (8, 9), designated ICEY, which is present only in strain GM274B.

A total of 33 surface lipoprotein genes were detected in the GM274B genome, but none were preceded by characteristic poly(TA) tract motifs that mediate phase-variable lipoprotein expression (10, 11) and that are identifiable in all sequenced genomes of *M. mycoides* cluster taxa. However, 28 predicted surface protein-encoding genes were preceded by a homopolymeric A or T tract (14 to 17 nucleotides in length), which may be mutationally exploited to accomplish variable patterns of antigen expression (12). Among the 46 genes encoding proteins containing the PARCEL domain (13), six are predicted lipoproteins, five contain frameshift mutations, and one is interrupted by ICEY insertion.

The genome presented herein is the first completely assembled example for the species and provides a reference for comparative genomics, as well as a chromosomal blueprint for a nonpathogenic mycoplasmal cloning host.

### Nucleotide sequence accession number. 

This complete genome sequence has been deposited at DDBJ/EMBL/GenBank under the accession number CP007520.
